# Non-invasive kinetic modelling approaches for quantitative analysis of brain PET studies

**DOI:** 10.1007/s00259-022-06057-4

**Published:** 2023-01-18

**Authors:** Chris W. J. van der Weijden, Pascalle Mossel, Anna L. Bartels, Rudi A. J. O. Dierckx, Gert Luurtsema, Adriaan A. Lammertsma, Antoon T. M. Willemsen, Erik F. J. de Vries

**Affiliations:** 1 Department of Nuclear Medicine and Molecular Imaging, University of Groningen, University Medical Center Groningen, Hanzeplein 1, 9713GZ Groningen, The Netherlands; 2 Department of Radiology, University of Groningen, University Medical Center Groningen, Hanzeplein 1, Groningen, The Netherlands; 3 Department of Neurology, University of Groningen, University Medical Center Groningen, Hanzeplein 1, Groningen, The Netherlands

**Keywords:** Positron emission tomography, Arterial input function, Image derived input function, Population-based input function, Simultaneous estimation of the input function

## Abstract

Pharmacokinetic modelling with arterial sampling is the gold standard for analysing dynamic PET data of the brain. However, the invasive character of arterial sampling prevents its widespread clinical application. Several methods have been developed to avoid arterial sampling, in particular reference region methods. Unfortunately, for some tracers or diseases, no suitable reference region can be defined. For these cases, other potentially non-invasive approaches have been proposed: (1) a population based input function (PBIF), (2) an image derived input function (IDIF), or (3) simultaneous estimation of the input function (SIME). This systematic review aims to assess the correspondence of these non-invasive methods with the gold standard. Studies comparing non-invasive pharmacokinetic modelling methods with the current gold standard methods using an input function derived from arterial blood samples were retrieved from PubMed/MEDLINE (until December 2021). Correlation measurements were extracted from the studies. The search yielded 30 studies that correlated outcome parameters (V_T_, DVR, or BP_ND_ for reversible tracers; K_i_ or CMR_glu_ for irreversible tracers) from a potentially non-invasive method with those obtained from modelling using an arterial input function. Some studies provided similar results for PBIF, IDIF, and SIME-based methods as for modelling with an arterial input function (*R*^2^ = 0.59–1.00, *R*^2^ = 0.71–1.00, *R*^2^ = 0.56–0.96, respectively), if the non-invasive input curve was calibrated with arterial blood samples. Even when the non-invasive input curve was calibrated with venous blood samples or when no calibration was applied, moderate to good correlations were reported, especially for the IDIF and SIME (*R*^2^ = 0.71–1.00 and *R*^2^ = 0.36–0.96, respectively). Overall, this systematic review illustrates that non-invasive methods to generate an input function are still in their infancy. Yet, IDIF and SIME performed well, not only with arterial blood calibration, but also with venous or no blood calibration, especially for some tracers without plasma metabolites, which would potentially make these methods better suited for clinical application. However, these methods should still be properly validated for each individual tracer and application before implementation.

**Supplementary Information:**

The online version contains supplementary material available at 10.1007/s00259-022-06057-4.

## Introduction

PET quantification with kinetic modelling is considered to be the best approach for quantitatively assessing tracer binding, being able to capture the early changes in biomarkers at disease onset, and during disease progression and treatment. The main parameters generated by kinetic modelling of PET data are net rate of influx (K_i_), volume of distribution (V_T_) and/or binding potential (BP_ND_). To measure these parameters, compartmental modelling with an arterial input function (AIF) is considered to be the gold standard, but graphical methods (e.g. Patlak [1] and Logan [2] graphical analyses) and basis function methods (e.g. spectral analysis [3]) in combination with an AIF are also commonly used methods for PET quantification. However, all these approaches are quite labour intensive and invasive [4, 5], as arterial blood sampling and metabolite analysis are required to obtain an AIF.

In some cases, obtaining an AIF might be difficult from a practical point of view. For example, in a total body PET scanner, the long line from the wrist to the site of sampling might cause excessive dispersion and delay effects and increase the risk of blood clotting. To circumvent arterial sampling, alternative methods for estimating K_i_, V_T_ and BP_ND_ without the need for an AIF have been developed, such as kinetic modelling methods using a reference tissue [6]. However, a reference region may not always be available, or assumptions underlying reference tissue models may not but valid, for example, when the blood–brain barrier (BBB) is locally disrupted by the disease.

Several alternative approaches to generate an input function have been proposed: i.e. the use of a population based (PBIF) [7], image derived (IDIF) [8] input function, and simultaneous estimation of the input function (SIME) [9]. These approaches are based on the non-invasive generation of an arterial input function rather than the identification of a reference region. The PBIF is based on a population based average of the AIF, while an IDIF is estimated from a region of interest (ROI) containing a vascular structure. SIME estimates the input function by extracting blood parameters from fitting time-activity curves (TACs) of multiple brain ROIs simultaneously.

The purpose of the present study was to provide an overview of published non-invasive alternatives to generate an input function and to assess their correlation and bias as compared with an (invasive) AIF. For simplicity purposes, the pharmacokinetic models applied are referred to as 1T2k for the reversible one-tissue compartment model and 2T3k and 2T4k for the irreversible and reversible two-tissue compartment models, respectively.

## Methods

### Search and selection procedure

Literature was screened according to the preferred reporting items for a systematic review and the meta-analysis for diagnostic test accuracy (PRISMA-DTA) statement using PubMed [10]. All relevant articles using either PBIF, IDIF or SIME for the analysis of human brain PET data until December 2021 were included without any language restrictions. The search string used is shown in the Supplementary information. Retrieved studies were assessed by two authors. Studies using a non-invasively generated input function for analysing cerebral PET data were only included if resulting kinetic parameters were compared with those obtained using an AIF. Studies that contained only in vitro, animal, phantom or simulated data and studies that lacked quantitative outcome parameters were excluded. The publication bias assessment can be found in the supplementary information.

### Assessment of the non-invasive kinetic modelling approaches

Literature was thoroughly assessed for the kind of methodology used (Supplementary information, Tables 1-3). The primary focus was on differences in the generation of the input functions. The correspondence of the alternative (non-invasive) input function was compared with the AIF generated after i.v. tracer injection by comparing the kinetic parameters (e.g. K_i_, V_T_ or BP_ND_) obtained from compartment modelling (i.e. 1T2k, 2T3k, 2T4k), linearization methods (e.g. Logan, Patlak) or basis function methods (e.g. spectral analysis). All studies correlating non-invasive kinetic modelling approaches with AIF-based kinetic modelling used either *R* or *R*^2^ values to describe the correspondence. For standardization purposes, *R* values were converted to *R*^2^. Bias was evaluated by assessing the slope and intercept of the correlation plot.

## Results of literature search

**Fig. 1:**
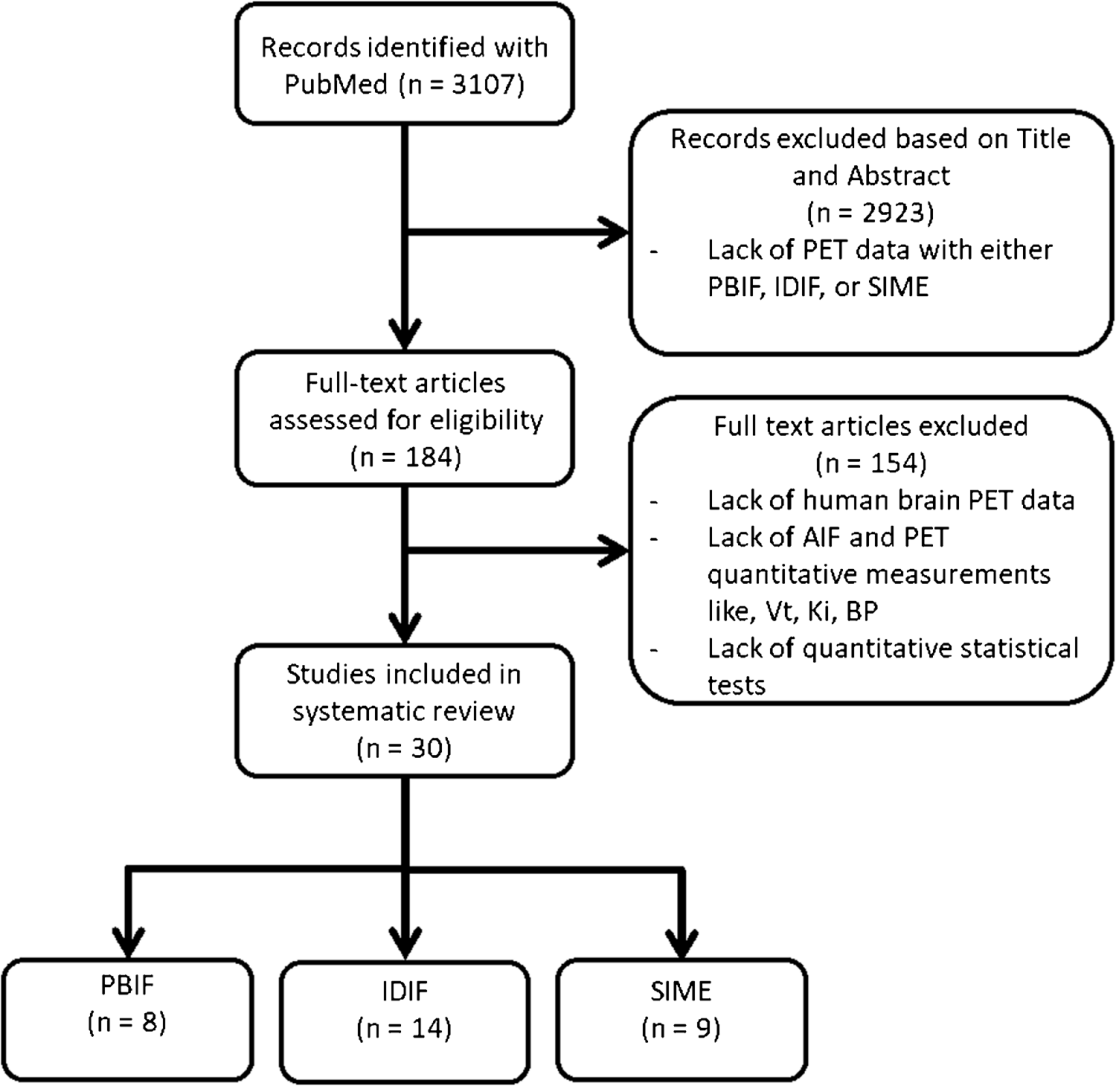
Literature assessment strategy employed

Screening of the literature resulted in 3107 unique articles (Fig. 1) of which 30 were actually included. One study [11] assessed both PBIF and SIME. Of the 30 studies, 8 assessed PBIF [7, 11–17], 14 IDIF [8, 18–30] and 9 SIME (Supplementary information, Table 4) [11, 31–40].

## Blood calibrations and metabolite corrections

The concentration of intact tracer in arterial plasma represents the true input function for kinetic modelling of PET data, provided that labelled metabolites do not cross the BBB. Traditionally, the arterial whole blood curve is acquired using either manual sampling or an automated online sampling device (sampler). When a sampler is used, some additional manual arterial blood samples are needed to determine the blood/plasma ratio. Using this ratio, the whole blood curve can be converted into an arterial plasma curve, which represents the total radioactivity concentration in plasma. If the plasma/blood ratio is stable over time, the average ratio from the manual samples can be used. Otherwise, the time course of the plasma/blood ratio can be fitted to a mathematical equation. Multiplying the whole blood curve with this equation provides the total plasma curve. Most PET tracers produce radioactive metabolites. Ideally, these metabolites do not cross the BBB, and, therefore, only the intact parent tracer should be considered the input function for modelling. In other words, the total plasma curve needs to be corrected for the presence of labelled metabolites. In practice, the manual samples mentioned above are also used to estimate the fraction of intact tracer in plasma over time, which again can be fitted to a mathematical equation. Multiplying the total plasma curve with this equation results in the metabolite corrected plasma curve that can be used as AIF. While the AIF is used to determine the biological response function, a whole blood curve is used to calculate the contribution of the blood compartment to the PET signal.

Non-invasive methods to estimate the AIF from a whole blood curve also require corrections for plasma/blood ratios and/or corrections for the presence of radio-labelled metabolites. This illustrates the main problem of non-invasive methods, as these corrections still require data from blood. As an alternative for arterial blood samples, both venous and arterialized venous blood samples have been proposed, in addition to using population based average data. Arterialized venous blood can be obtained by heating the extremity of the patient, resulting in venous blood being more similar to arterial blood [41–43]. Blood samples are generally obtained more than 10 min after tracer injection, when differences between arterial and venous blood should be small.

## Population-based input function

For PBIF-based quantification methods, blood data from a representative set of subjects (population) are used to create an AIF, which in turn is used for kinetic modelling of PET data from an individual subject. Whole blood, plasma or metabolite corrected plasma TACs of multiple subjects can be used to generate a PBIF. The generation of a PBIF generally starts with shifting individual curves to align their peaks. These curves are then calibrated using, for example, injected dose, body surface area, body weight or lean body mass. Subsequently, the resulting curves are averaged providing a mean population based AIF, i.e. the PBIF. Finally, for each new individual subject, this PBIF is then calibrated using the same parameter(s) as mentioned above to obtain a normalised PBIF for that subject. In most studies, one or more manual blood samples are also used in the calibration process.

### Correspondence of population based input function methods with the gold standard

#### [^18^F]FDG

The first article comparing PBIF and AIF based methods was published by Takikawa et al. in 1993 (Table 1 and Supplementary information Table 1) [7]. In this [^18^F]FDG study (*n* = 34), a good correlation (*R*^2^ = 0.98, slope = 0.99) was found between CMR_glu_ estimated using an AIF and a PBIF with arterial blood calibration. A PBIF with arterial blood calibration was also used to evaluate [^18^F]FDG data in a study by Roccia et al. [11]. However, in this study on patients with Alzheimer’s disease (AD) and mild cognitive impairment (MCI), only a moderate correlation was found (*R*^2^ = 0.59, *n* = 49). Differences were observed in the number of arterial blood calibration samples. However, the main explanation for the discrepancy between studies might be the used validation method. Unfortunately, Roccia et al. did not report their validation strategy, while Takikawa et al. based their PBIF on 10 subjects and used 24 subjects to validate it. In addition, Roccia et al. also did not report the slope or intercept of the correlation plot, whereas Takikawa reported a slope of 0.99, indicating that modelling of [^18^F]FDG data with a PBIF using arterial blood samples for calibration can be performed with low bias. However, given the low number of studies, further validation studies are required.

**Table 1:** Correspondence of the results of various PBIF approaches with those derived from AIF-based pharmacokinetic modelling

Study	Tracer	Blood calibration	Metabolite correction	Kinetic model	Outcome parameter	Correlation (*R*^2^)	Bias (slope; [intercept])
Roccia, 2019 [11]	[^18^F]FDG	Arterial	No	2T3k	CMR_glu_	0.59	n.r
Takikawa, 1993 [7]	[^18^F]FDG	Arterial	No	2T3k	CMR_glu_	1.00	0.99
McGinnity, 2018 [13]	[^18^F]GE179	Arterial	Yes	Spectral analysis	V_T_	0.81	1.02 [-0.20]
Lavisse, 2015 [14]	[^18^F]DPA714	Arterial	Yes	2T4k (AIF) Logan (PBIF)	V_T_	0.92	n.r
Lavisse, 2015 [14]	[^18^F]DPA714	Venous	Yes	Logan	V_T_	0.45 (mean)	n.r
Mabrouk, 2017 [15]	[^18^F]FEPPA	Arterial	Yes	2T4k (AIF)	V_T_	0.86	n.r
				Logan (PBIF)			
Rissanen, 2015 [16]	[^11^C]TMSX	Arterial	Yes	Logan	V_T_	0.94	n.r
					DVR	0.67–1.00 (range)	
Rissanen, 2015 [16]	[^11^C]TMSX	None	Population	Logan	V_T_	0.58	n.r
					DVR	0.67–1.00 (range)	
Zanotti-Fregonara, 2013 [17]	[^18^F]FMPEP-d2	Arterial	Yes	Logan	V_T_	0.88	n.r
Takikawa, 1994 [12]	[^18^F]FDOPA	Arterial	Yes	2T3k	K_i_	0.98	n.r

AIF = arterial input function, PBIF = population-based input function, CMR_glu_ = cerebral metabolic rate of glucose, V_T_ = volume of distribution, DVR = distribution volume ratio, K_i_ = net influx rate, 2T3k = irreversible two tissue compartment model, 2T4k = reversible two tissue compartment model, n.r. = not reported

#### Neuroinflammation tracers

The use of a PBIF has also been investigated for kinetic modelling of several neuroinflammation tracers that bind to the 18 kD translocator protein (TSPO; Table 1 and Supplementary information Table 1). Lavisse et al. (2015) analysed the use of a PBIF with arterial blood calibration and metabolite correction for quantification of [^18^F]DPA714 in 10 healthy controls (HC) and found a good correlation (*R*^2^ = 0.92) with the outcome parameters obtained using an AIF. Interestingly, they found only a moderate correlation (*R*^2^ = 0.45) when a PBIF with venous blood calibration and metabolite correction was used [14]. Potential reasons for this difference are unknown. Lavisse et al. did not report the individual metabolite fractions in arterial and venous samples. McGinnity et al. (2018) found that kinetic modelling of [^18^F]GE179 with a PBIF in 9 epilepsy patients and 11 HC correlated well with the AIF-based approach (*R*^2^ = 0.81) and showed little bias (slope = 1.02, intercept =  − 0.20), if the PBIF was calibrated and metabolite corrected with arterial blood samples [13]. Mabrouk (2017) assessed the use of a PBIF with arterial blood calibration and metabolite correction for the quantification of [^18^F]FEPPA in 39 HC, 18 AD and 16 Parkinson’s disease (PD) patients and showed good correlations (*R*^2^ = 0.98) between the V_T_ obtained using an AIF and the PBIF [15].

#### Other tracers

Rissanen et al. (2015) investigated whether a PBIF with or without arterial blood calibration and metabolite correction could be used as input function for kinetic modelling of [^11^C]TMSX data of 7 HC, 12 multiple sclerosis (MS) patients, and 9 PD patients [16]. Quantification using the calibrated and metabolite corrected PBIF showed high correlation (*R*^2^ = 0.94) as compared with AIF-based quantification, but the non-calibrated PBIF did not (*R*^2^ = 0.58). Furthermore, Zanotti-Fregonara et al. (2013) found a high correlation (*R*^2^ = 0.88) when quantifying [^18^F]FMPEP-d2 data of 42 HC using a PBIF calibrated and metabolite corrected with arterial blood samples as compared with quantification using an AIF [17]. Finally, Takikawa et al. (1994) investigated the use of a PBIF calibrated and metabolite corrected with an arterial blood sample as input function for kinetic modelling of [^18^F]FDOPA data of 12 HC and 12 PD patients and found an excellent correlation (*R*^2^ = 0.98) with AIF derived results [12].

### Concluding remarks on the use of a PBIF

In general, use of arterial samples for PBIF calibration and metabolite correction leads to more accurate measurements of kinetic parameters than use of venous samples or use of a PBIF without blood correction at all, although comparative studies are scarce. Discrepancies between individuals in tracer clearance, tracer extraction, blood velocity and metabolism are among the main aspects hampering proper implementation of a PBIF without blood calibration and metabolite correction, and therefore at least 1 blood sample to calibrate the plasma curve and correct for metabolites is still required. This blood sample is preferably drawn after peak activity has passed and the blood radioactivity concentration has become more stable, which is often later than 30 min after tracer injection. In addition, the PBIF could be affected by disease or intervention, and therefore a specific PBIF should be generated for each condition, unless it has been proven that the PBIF is not affected.

## Image-derived input function

An IDIF is an input function, which can be generated by placing an ROI over a suitable vascular structure in the PET scan. In case of brain PET studies, usually the carotid arteries are used. These are visible in early timeframes immediately after tracer injection, when the radiotracer is still restricted to the blood pool. Another method to delineate the blood pool is the use of magnetic resonance imaging (MRI) or CT angiography to delineate the blood vessels and transfer these ROIs to the PET scan of the same individual. There are also statistical approaches to extract blood data from the PET images, like independent component analysis [29]. This approach is based on the fact that blood TACs have a completely different shape than tissue TACs, regardless of the underlying kinetic properties of the tracer. After delineation of the blood pool via any of these methods, a time activity curve is created representing the whole blood signal within the PET scan. However, also for IDIF, the challenge remains to generate a metabolite corrected plasma curve from a whole blood curve. Therefore, use of one or more arterial or venous blood samples is still required to calibrate the whole blood curve, determine the blood-plasma ratio and correct for radioactive metabolites.

### Validation of the image-derived input function

Among the non-invasive methods to create an input function, the IDIF should be the most accurate one, at least in theory, as the blood curve is measured directly from the PET images and therefore should accurately represent tracer activity in blood. In fact, if sufficiently short time frames (e.g., ≤ 10 s) are selected during the first minutes of the PET acquisition, the whole blood curve obtained using an IDIF is, in general, sharper than the whole blood curve measured using arterial blood sampling, as it is not affected by dispersion in the arterial cannula and external lines [44]. A slower injection protocol will most likely result in an IDIF more similar to the AIF. However, a slower injection will negatively affect the robustness of the microparameter estimates, which can be more reliably estimated with a bolus injection. The accuracy of the IDIF-based quantification method is, however, dependent on several aspects, like the intrinsic spatial resolution of the PET scanner, the reconstruction algorithm used and the definition of the ROIs. The carotid arteries are the main arteries within the field-of-view of most PET scanners (typically ~ 25 cm). They have a diameter of ~ 5 mm, which is of the same order of magnitude as the resolution of most scanners. Small structures, like carotids, are therefore prone to partial volume effects, i.e. blood-to-tissue spill-out effects during the early phase and tissue-to-blood spill-in effects during the late phase of the PET scan. Typically, a partial volume correction (PVC) method is applied to limit these effects [45]. However, it still is not clear which PVC would be best for the carotid arteries. Furthermore, small ROIs, like the carotid arteries, result in a lower signal-to-noise ratio and thus poorer precision. With the introduction of large field-of-view PET/CT cameras, it is possible to have both the heart and the brain within the field of view, which enables the generation of IDIF curves for PET studies of the brain using the heart or aorta as blood pool [46, 47]. This would lead to more accurate IDIF curves, as both ventricles and aorta have a much larger diameter than the carotid arteries. Besides the resolution of the PET camera and the generation of the ROI, the applied reconstruction parameters also have a large effect on the accuracy of the IDIF, because the reconstruction parameters can affect the signal-to-noise ratio and determine the time frames used to generate the input function. For example, too many iterations increase the noise, whereas too few iterations reduce the contrast in the images, leading to difficulties obtaining the blood peak. In addition, short time frames are required to accurately capture the blood peak (preferably ≤ 10 s), as longer frame times lower the amplitude and broaden the blood peak by averaging the peak activity over a longer period. However, these early time frames should not be too short, as the small number of coincidences detected in such a short time frame would lead to a poor signal-to-noise ratio. Longer frame times can be used after blood peak has passed and blood activity levels change more slowly. For these reasons, the validation of IDIF-based quantification methods theoretically has to be performed per scanner, since the scanner resolution has a large impact on partial volume effects and the optimal number of iterations, and therefore the accuracy of the obtained blood TACs.

### Correspondence of the image derived input function with the gold standard

#### [^18^F]FDG

So far, two studies [18, 23] (*n* = 9, 16) investigated the use of an IDIF with arterial blood calibration as input function for kinetic analysis of [^18^F]FDG data, three [8, 24, 25] investigated the use of an IDIF with venous blood calibration (*n* = 16, 22, 26), and one [26] used an IDIF without any blood calibration (*n* = 6, Table 2). Both studies comparing kinetic results between IDIF with arterial blood calibration and AIF showed high correlations (*R*^2^ = 0.85–1.00) and low bias (slope = 0.92–1.09, intercept = 0.00–0.00) [18, 23]. Noteworthy is that the variation in correlation coefficients seemed to be caused primarily by the quantification method employed, as they were highly comparable (*R*^2^ = 0.99–1.00, slope = 0.91–1.09) when Patlak analysis was used, but lower for non-linear regression (*R*^*2*^ = 0.85, slope = 0.97) [23]. The three studies investigating the use of an IDIF with venous blood calibration also found high correlation (*R*^2^ = 0.98–1.00) and low bias (slope = 0.92–1.11, intercept = – 0.04–0.05) when compared with AIF results [8, 24, 25]. Even pharmacokinetic modelling of [^18^F]FDG data using an IDIF without blood calibration showed high correlation (*R*^2^ = 0.91), although this study included only 6 subjects and the bias was not reported [26]. All studies mentioned above only included HC, so the impact of disease cannot be assessed yet.

**Table 2:** Correspondence of the results of various IDIF approaches with those derived from AIF-based pharmacokinetic modelling

Study	Tracer	Blood calibration	Metabolite correction	Kinetic model	Outcome parameter	Precision (*R*^2^)	Bias (slope; [intercept])
Zhou 2012 [18]	[^18^F]FDG	Arterial	No	Patlak	K_i_ (ROI based)	0.99–1.00	0.92–1.09 [0.00–0.00]
					K_i_ (voxel-based)	0.99–1.00	0.92–1.08 [0.000.00]
Huisman 2012 [23]	[^18^F]FDG	Arterial	No	Patlak	K_i_	0.99 ± 0.01 (mean)	0.99 ± 0.06 (mean)
				NLR	K_i_	0.85 ± 0.34 (mean)	0.97 ± 0.13 (mean)
Zhou 2011 [24]	[^18^F]FDG	Venous	No	Patlak	K_i_ (ROI based)	0.99–1.00	1.00–1.09 [0.00–0.00]
					K_i_ (voxel-based)	0.98–1.00	1.00–1.11 [0.00–0.00]
Chen 1998 [8]	[^18^F]FDG	Venous	No	Patlak	CMRglc	0.99–1.00	0.92–1.09 [– 0.04–0.05]
Chen 2007 [25]	[^18^F]FDG	Venous	No	Patlak	CMRglc	0.99–1.00	1.02 [0.00]
Croteau 2010 [26]	[^18^F]FDG	None	No	2T3K	CMR_glc_	0.91	n.r
Galovic, 2021 [20]	[^18^F]GE179	Venous	Yes	2T4k	V_T_	0.90	n.r
Mabrouk 2014 [27]	[^18^F]FEPPA	Arterial	Yes	2T4K	V_T_	0.70–0.87	0.83–1.01
					V_T_ (ICA)	0.88–0.92	1.01–1.17
Zanderigo 2018 [28]	[^11^C]Arachidonic acid	None	Population based	2T3K	K_i_	0.84	1.06 [0.31]
Mertens, 2021 [22]	[^18^F]JNJ-64413739	Arterial	Yes	Logan	V_T_	0.69	n.r
Schain 2013 [29]	[^11^C]Flumazenil	Arterial	Yes	Logan	V_T_ (MDC)	0.98	1.21 [– 0.17]
					V_T_ (4HVC)	0.95	1.29 [– 0.45]
					V_T_ (PWC)	0.96	1.10 [– 0.13]
Schain 2013 [29]	[^11^C]AZ10419369	Arterial	No	Logan	V_T_ (MDC)	0.89	1.30 [– 0.25]
					V_T_ (4HVC)	0.90	1.35 [– 0.23]
					V_T_ (ICA)	0.90	1.29 [– 0.21]
					V_T_ (PWC)	0.96	1.01 [– 0.02]
Bahri 2017 [30]	[^18^F]UCB-H	Arterial	Yes	Logan	V_T_	> 0.99 (mean)	0.98–1.06 [–0.02–0.04]
Islam 2017 [19]	[^15^O]H_2_O	None	No	1T2K	K_1_	> 0.99	1.00 [0.05]
Vestergaard, 2021 [21]	[^15^O]H_2_O	None	No	1T2k	K_1_	0.87	n.r

V_T_ = volume of distribution, ROI = region of interest, CMR_glc_ = cerebral metabolic rate of glucose, ICA = independent component analysis, 1T2k = reversible one tissue compartment model, 2T3k = irreversible two tissue compartment model, 2T4k = reversible two tissue compartment model, 4VHC = 4 hottest voxels of carotids, MDC = manual delineation of carotids, PWC = pairwise correlation, n.r. = not reported, NLR = nonlinear regression

#### Neuroinflammation tracers

The use of an IDIF for quantification of the neuroinflammation tracers [^18^F]GE179, [^18^F]FEPPA (all TSPO), [^11^C]arachidonic acid (cyclooxygenase) and [^18^F]JNJ-64413739 (P2X7 receptors) has also been investigated (Table 2 and Supplementary information Table 2). Remarkably, all these studies only included healthy subjects, who supposedly should not suffer from neuroinflammation. Consequently, the impact of inflammation induced perfusion effects cannot be assessed [48]. In addition, the use of HC alone, and thus regions with low specific uptake, means that correlations have been performed only with low V_T_ values, and it therefore remains unclear whether correlations remain the same in the presence of a high specific signal. The use of an IDIF with venous blood calibration and metabolite correction for kinetic analysis of [^18^F]GE179 data resulted in high correlation (*R*^2^ = 0.90, *n* = 10) when compared with AIF based results [20]. Unfortunately, the bias was not reported in this study. For quantitative analysis of [^18^F]FEPPA, Mabrouk et al. used independent component analysis to derive the IDIF [27], which was subsequently calibrated and metabolite corrected using arterial blood samples. This approach produced outcome parameters that correlated better (*R*^2^ = 0.88–0.92, slope = 1.01–1.17, *n* = 18) with those derived from AIF-based analyses than using an IDIF generated from an ROI that was manually delineated on the internal carotid (*R*^2^ = 0.70–0.87, slope = 0.83–1.01, *n* = 18). While for all TSPO tracers there is endothelial binding, which increases with the stronger tracer affinity for TSPO [49]. Binding of the tracer to endothelial cells might theoretically cause an overestimation of the image derived whole blood curve and consequently of the IDIF. However, the high correlations observed between the 2T4k V_T_ using an AIF and the 2T4k V_T_ using an IDIF for [^18^F]GE179 and [^18^F]FEPPA suggest that the influence of endothelial TSPO binding is negligible. Zanderigo et al. only used a population based metabolite correction of the IDIF for the analysis of [^11^C]arachidonic acid PET scans and found better correlations between IDIF and AIF-based results (*R*^2^ = 0.84, slope = 1.06, intercept = 0.31, *n* = 11) than when an IDIF uncorrected for metabolites was used [28]. Use of an IDIF with arterial blood calibration and metabolite correction for kinetic analysis of [^18^F]JNJ-64413739 data showed a correlation of *R*^2^ = 0.69 (*n* = 11) compared with AIF based results [22]. These studies suggest that the use of an IDIF for neuroinflammation tracers might be feasible in some cases, but still requires substantial validation, especially in patients suffering from neuroinflammation.

#### Other tracers

A study by Schain et al. (2013) found that kinetic analysis of [^18^F]flumazenil data using an IDIF, calibrated and metabolite corrected with arterial plasma samples, highly correlated with AIF-based analysis (*R*^2^ = 0.95–0.98, *n* = 6), but showed a moderate bias (slope = 1.10–1.29, intercept = – 0.45–0.13) [29]. Schain et al. (2013) also investigated the use of an IDIF with arterial blood calibration and metabolite correction for quantifying [^11^C]AZ10419369 data of 6 HC and again found a high correlation (*R*^2^ = 0.89–0.96) with AIF-based modelling and a moderate bias (slope = 1.01–1.35, intercept = – 0.25–0.02) [29]. Bahri et al. (2017) investigated the use of an IDIF with arterial blood calibration and metabolite correction as input function for kinetic analysis of [^18^F]UCB-H data and found high correlation (*R*^2^ > 0.99) and low bias (slope = 0.98–1.06, intercept = – 0.02–0.04), but this study included only 4 HC [30]. Islam et al. (2017) and Vestergaard et al. (2021) investigated the use of an IDIF for analysing [^15^O]H_2_O data of patients with cerebrovascular disease and HC [19, 21]. As [^15^O]H_2_O does not produce metabolites, the IDIF was used without blood calibration. Islam et al. (2017) found higher correlations than Vestergaard et al. (2021) (*R*^2^ > 0.99, *n* = 33 vs. *R*^2^ = 0.87, *n* = 19) [19, 21]. Vestergaard et al. (2021) ascribed the discrepancy between K_1_ values measured with AIF and IDIF to inaccuracies in the measurement of the AIF, but they also found that only a moderate reproducibility in K_1_ estimates when an IDIF was used [21]. This difference in correlation between both studies could also be due to differences in ROI delineation of the internal carotid artery, as the ROI for the blood pool was defined on the PET images by Islam et al. and on the MRI scan by Vestergaard et al.

### Concluding remarks on the use of an IDIF

The results of IDIF based pharmacokinetic modelling showed moderate to excellent correlations with the outcome of AIF-derived quantification. However, most studies only included HC and used arterial blood samples for calibration and metabolite correction of the IDIF. Nevertheless, pharmacokinetic modelling of [^15^O]H_2_O and [^18^F]FDG data with an IDIF without any blood calibration showed high correlations and low bias and thus may be used to substitute kinetic modelling with an AIF [19, 26]. In addition, the use of an IDIF with venous calibration also seemed to perform well for [^18^F]FDG [8, 24, 25]. This is most likely because differences in whole blood and plasma have little impact on the estimation of the [^18^F]FDG macroparameters [8], as the blood-plasma ratio is constant over time during a scan of 60 min and has limited intersubject variation. Nonetheless, for determination of CMR_glu_, a venous blood sample is still required for determining the glucose concentration in blood when converting K_i_ to CMR_glu_. For other tracers than [^15^O]H_2_O and [^18^F]FDG, the performance of IDIF using venous samples was poor, whereas the application of arterial blood samples for calibration and metabolite correction undermined the main purpose of using an IDIF, i.e. to reduce the invasiveness of the procedure.

## Simultaneous estimation of the input function

The tracer tissue concentration as measured with PET is determined by the AIF and the (physiological) impulse response function [31, 50]. The rate constants of the impulse response function can be estimated by fitting the tracer concentration measured with PET (which is composed of both signal from tissue and blood) to an appropriate compartment model with the AIF as input function. When the optimal compartment model for the tracer of investigation is defined a priori, a series of distinct tissue TACs can be used to extract the parent plasma TAC by fitting all TACs simultaneously. For every tissue TAC, the solution for the AIF must be the same, as the tracer concentration in plasma is considered to be the same across the brain. The use of multiple tissue TACs reduces the number of possible solutions for the AIF. Therefore, an increase in the number of TACs and the use of TACs with sufficient diversity in shape (reflecting different tracer kinetics) enables the estimation of the AIF, at least in theory.

Several confounders affect the generation of an input function by SIME, such as patient motion or subtle measurement errors (noise) in the later time frames of the PET scan. These confounders can affect the approximation of individual rate constants, and consequently the SIME generated AIF. From a theoretical point of view, SIME could accurately denominate the plasma concentration, but in practise, there are too few TACs to correctly solve the formulas, because there are too many unknown variables. To compensate for the computational complexity, a blood sample can be taken to determine the parent tracer concentration in plasma, which can be used to scale the plasma concentration curve of SIME appropriately to the AIF.

### Validation of simulation estimation of the input function

The fundamental principles of SIME were derived in 1994 [50]. The renewed interest in this method is mainly due to recent technological advances in computer speed, reducing computational time significantly. The accuracy of SIME highly depends on both the number and the diversity in shapes of the tissue TACs used [51]. The acquisition of sufficiently divergent TACs is generally considered to be the main problem with SIME. Nonetheless, the number of tissue TACs is generally restricted in order to reduce computational time. Therefore, often first a selection is made of differently shaped tissue TACs through visual inspection. To reduce the computational complexity of SIME, the true AIF is often substituted for a model to reduce the number of unknown variables. However, SIME has difficulties with accurately estimating the variables that describe the tail of the curve [52]. The integration of blood data describing the tracer parent fraction concentration in plasma within the models of the plasma curve reduces further the computational complexity of SIME and thereby enhances the accuracy of parameter estimation [33, 52]. The application of a 3-exponential model for the plasma curve in SIME results in better estimates of the tail than the peak of the AIF. Although this has minimal effect on estimated macroparameters, it reduces accuracy of estimated individual microparameters [31]. The advantage of SIME is that it generates an AIF from the images, irrespective of the PET scanner resolution and that it is specific for the individual.

### Correspondence of SIME with the gold standard

#### [^18^F]FDG

Two studies have been retrieved that used arterial blood and one study that used venous blood for the calibration of the SIME derived [^18^F]FDG input function, and one study did not use any blood calibration at all (Table 3). When the SIME derived input function was calibrated with an arterial blood sample, the two studies showed quite discrepant correlation (*R*^2^ = 0.72–0.88, *n* = 49 vs. *R*^2^ = 0.91–0.95, *n* = 9), despite hardly any methodological differences between the two studies [11, 31]. Both studies used the 2T3k model for quantification, a single blood sample collected at 40 min and the same ROIs. The main difference was the sample size (49 versus 9). Therefore, it seems likely that the small sample size may not be representative for the general population. The study, in which the SIME derived input function was calibrated with venous samples, showed both high correlation (*R*^2^ = 0.94, *n* = 3) and low bias (slope = 1.01, intercept = 0.01) [33]. However, it should be noted that only 3 subjects were included in this study. The study using SIME without blood calibration resulted in only moderate correlation (*R*^2^ = 0.62–0.69, *n* = 49) [11]. This indicates that the scaling of SIME with a blood sample seems to perform better for accurate and precise quantification of [^18^F]FDG data. This might be due to inaccuracies caused by the blood volume in the absence of blood calibration, noise or the lack of a blood sample that requires an increased number of TACs. Whether venous samples can be used for this purpose remains to be confirmed.

**Table 3:** Correspondence of the results of various SIME approaches with those derived from AIF-based pharmacokinetic modelling

Study	Tracer	Blood calibration	Metabolite correction	Kinetic model	Outcome parameter	Correlation (*R*^2^)	Bias (slope; intercept)
Roccia, 2019 [11]	[^18^F]FDG	Arterial	No	2T3K	CMR_glu_	0.72–0.88	n.r
Ogden, 2010 [31]	[^18^F]FDG	Arterial	No	2T3k	K_i_	0.91–0.95	0.70–0.88 [– 0.00–0.01]
Wong, 2001 [33]	[^18^F]FDG	Venous	No	2T3k	CMR_glu_	0.94	1.01 [0.01]
Roccia, 2019 [11]	[^18^F]FDG	None	No	2T3K	CMR_glu_	0.62–0.69	n.r
Schain, 2018 [34]	[^11^C]PBR28	Arterial	Yes	2T4k	BP_ND_ (AIF)	0.56	n.r
					V_T_ /fp (SIME)		
Schain, 2018 [34]	[^11^C]PBR28	None	Template	2T4k	BP_ND_	0.78	0.92 [0.10]
					BP_ND_ (AIF)		
					V_T_ /fp (SIME)	0.37	n.r
Bartlett, 2019 [35]	[^11^C]CUMI	Arterial	Yes	LEGA	V_T_	0.81	0.92 [0.76]
					BP_ND_		
Zanderigo, 2015 [36]	[^11^C]CUMI	Arterial	Yes	LEGA	V_T_	0.84	0.93 [1.58]
					BP_ND_	0.90	0.99 [0.64]
Bartlett, 2019 [35]	[^11^C]CUMI	Venous	Yes	LEGA	V_T_	0.36	0.79 [5.52]
Zanderigo, 2015 [36]	[^11^C]CUMI	None	Predicted	LEGA	V_T_	0.69	0.72 [4.07]
					BP_ND_	0.78	0.84 [1.60]
Ogden, 2010 [31]	[^11^C]DASB	Arterial	Yes	1T2k	V_T_	0.95	0.84 [2.96]
Bartlett, 2019 [35]	[^11^C]DASB	Arterial	Yes	LEGA	V_T_	0.79	0.84 [6.90]
					BP_ND_		
Bartlett, 2019 [35]	[^11^C]DASB	Venous	Yes	LEGA	V_T_	0.68	0.68 [4.12]
Mikhno, 2015 [37]	[^11^C]DASB	None	Predicted	1T2k	V_T_	0.77	n.r
					BP_ND_	0.92	
Ogden, 2010 [31]	[^11^C]WAY-100635	Arterial	Yes	2T4k	V_T_	0.82	1.00 [0.01]
Sari, 2018 [38]	[^11^C]SB201745	None	Fitted	2T4k	V_T_	0.94	n.r
					BP_ND_	0.97	
Bartlett, 2019 [35]	[^11^C]ABP688	Arterial	Yes	2T4k	V_T_	0.79	0.82 [0.76]
Bartlett, 2019 [35]	[^11^C]ABP688	Venous	Yes	2T4k	V_T_	0.87	1.08 [0.17]
Zanderigo, 2018 [32]	[^11^C]Harmine	Arterial	Yes	1T2k	V_T_	0.90	1.07 [– 1.04]
						0.88–0.93 (ROI wise)	1.11–1.20 [– 4.35 to – 0.59]
Ogden, 2010 [31]	[^11^C]BTA	Arterial	Yes	2T4k	V_T_	0.85	1.03 [0.08]

LEGA = likelihood estimation in graphical analysis, AIF = arterial input function, BP_ND_ = binding potential, V_T_ = volume of distribution, CMR_glc_ = cerebral metabolic rate of glucose, K_i_ = net influx rate, 1T2k = reversible one tissue compartment model, 2T3k = irreversible two tissue compartment model, 2T4k = reversible two tissue compartment model, n.r. = not reported

#### Neuroinflammation tracers

[^11^C]PBR28 is the only neuroinflammation tracer for which kinetic modelling with SIME has been investigated (Table 3). When a SIME derived input function with arterial blood calibration and metabolite correction was used as input for kinetic analysis of [^11^C]PBR28 data from 21 AD patients and 15 HC, only moderate correlation (*R*^2^ = 0.56) as compared with AIF based results was obtained [34]. Similarly, the application of SIME without blood calibration and metabolite correction, but with the use of a population based template, yielded only a moderate correlation (*R*^2^ = 0.37–0.78), although with low bias (slope = 0.92, intercept = 0.10) [34]. The moderate correlations of SIME using both arterial blood calibration and a population–based calibration might be due to limited divergence in TACs, which could be either due to the tracer kinetics or due to limited neuro-inflammation (and thus limited specific uptake) within the studied subjects.

#### Other tracers

Interestingly, good correlation without taking any blood samples could be achieved for the tracer [^11^C]SB201745 (*R*^2^ = 0.94–0.96, *n* = 6) despite rapid metabolism [38]. For [^11^C]SB201745, the excellent correlation could be achieved by incorporation of an IDIF and Hill function for metabolite estimation, which significantly reduced the computational complexity of SIME [38]. This combination of SIME with an image-derived whole blood curve together with a Hill function for metabolite estimation also improved the estimation of the shape of the AIF curve including the peak (Supplementary information Table 3) [38]. SIME using arterial blood calibration and metabolite correction for [^11^C]DASB quantification resulted in high correlation (*R*^2^ = 0.95, *n* = 25) for 1 study and in moderate correlation (*R*^2^ = 0.77, *n* = 18) for another, while both had comparable moderate bias (slope = 0.84) [31, 35]. However, application of venous blood calibration and metabolite correction resulted in moderate correlation and bias (*R*^2^ = 0.68, slope = 0.68, *n* = 18) [35]. For [^11^C]DASB, the non-invasive SIME approach had good correlations (*R*^2^ = 0.77–0.92, *n* = 96), making use of machine learning to determine which electronic health records (EHR) variables are best suitable for predicting the AIF [37]. The extracted variables were related to heart rate, blood pressure and body size, and apparently these variables are good enough to enable the estimation of a metabolite corrected plasma curve. This is especially surprising as it seems to outperform SIME with blood calibrations and metabolite corrections, at least for [^11^C]DASB. SIME with arterial, venous or no blood calibration and metabolite correction has also been applied for quantification of [^11^C]CUMI data. However, none of the methods seemed to be precise (*R*^2^ = 0.81–0.90, slope = 0.92–0.99, intercept = 0.64–1.58, *n* = 14–19; *R*^2^ = 0.36, slope = 0.79, intercept = 5.52, *n* = 19; *R*^2^ = 0.69–0.78, slope = 0.72–0.84, intercept = 1.60–4.07, *n* = 14; respectively) enough to substitute the AIF [35, 36]. Interestingly, [^11^C]CUMI BP_ND_ could be estimated with somewhat higher correlation and bias than V_T_, irrespective whether SIME was calibrated and metabolite corrected with an arterial blood sample or with a predicted metabolite correction [42]. Application of SIME, calibrated and metabolite corrected with an arterial blood sample, to quantify [^11^C]WAY-100635, [^11^C]BTA or [^11^C]ABP688 data resulted in estimations of V_T_ with fairly good correlation (*R*^2^ = 0.79–0.85, *n* = 7–10). Although bias of [^11^C]ABP688 V_T_ was only moderate (slope = 0.82, intercept = 0.76), it was good for [^11^C]WAY-100635 (slope = 1.00, intercept = 0.01) and [^11^C]BTA (slope = 1.03, intercept = 0.08)[37,41]. Calibration and metabolite correction of SIME with a venous blood sample slightly improved the quality of [^11^C]ABP688 V_T_ estimates (*R*^2^ = 0.87, slope = 1.08, intercept = 0.17), although this effect may be due to the relatively small sample size (*n* = 10) [41]. When SIME with arterial blood calibration and metabolite correction was used to estimate [^11^C]harmine V_T_ in 5 HC, good correlation was obtained (*R*^2^ = 0.88–0.93), but bias was poor (slope = 1.11–1.20, intercept = – 4.35 to – 0.59) [38].

### Concluding remarks about the use of SIME

The correspondence of SIME was highly variable across studies, irrespective of the method used for blood calibration and metabolite correction. One of the factors that may have affected the results of SIME is the outcome parameter used. In contrast to the studies investigating the PBIF and IDIF, various studies on SIME used the BP_ND_ as outcome parameter. Estimation of BP_ND_, calculated as the k_3_/k_4_ ratio, is notoriously prone to high standard errors. Therefore, poor correlations between BP_ND_ estimated with SIME and AIF-based compartment modelling may be due to unreliable AIF based BP_ND_ estimates, which would underestimate the performance of SIME. A limited number of studies showed positive results for the quantification of [^11^C]SB201745 and [^11^C]DASB data using a completely non-invasive SIME. SIME without blood calibration provided disappointing results for [^18^F]FDG, but SIME with either arterial or venous blood calibration worked well. The non-invasive SIME approaches for [^18^F]FDG data used machine learning to predict the [^18^F]FDG plasma fraction from EHR-derived variables. The negative results of these attempts suggest that the [^18^F]FDG plasma fraction could not be accurately predicted, which is surprising as the plasma/blood ratio for [^18^F]FDG has low intersubject variability and no metabolites are formed. Perhaps the use of other variables would make it more accurate, but nonetheless, for [^18^F]FDG, SIME with venous blood calibration showed promise in substituting the AIF [11]. For other tracers than [^18^F]FDG, SIME does not perform good enough in either a non-invasive or less-invasive form to substitute AIF. Even for [^18^F]FDG, the non-invasive or less-invasive SIME methods have been shown to perform well in only one study with small sample sizes, which further emphasizes the need for further validation of this method.

## Discussion

The aim of this study was to assess the correspondence of potentially non-invasive methods to generate an input function (PBIF, IDIF, SIME) for kinetic analysis of brain PET data with the gold standard, i.e. arterial sampling. The main methodological variations in the various approaches were whether manual blood samples were used for calibration and metabolite correction of PBIF, IDIF or SIME and, if so, whether these samples were arterial, arterialised venous or venous. Most studies that made use of arterial calibration and metabolite correction showed promising results, but such methods cannot really be considered as less- or non-invasive methods, as they still require the collection of arterial blood samples. The correspondence of these non-invasive methods was determined by comparing estimated macroparameters with those obtained using an AIF. Overall, this correspondence was moderate to excellent. Furthermore, it seems that, for optimal performance, PBIF requires calibration and metabolite correction with arterial blood samples, while IDIF and SIME seem to be promising non-invasive quantification methods, especially for [^18^F]FDG.

Although almost every study described the use of arterial sampling in their methodology, only a few actually investigated full kinetic analysis using a non-invasively generated input function in comparison with compartment modelling using an AIF. Most studies used a linearization method for comparing V_T_ or K_i_ estimates. Although these linearization methods are generally well established methods, they should be carefully implemented, as they are prone to bias. Due to physiological assumptions in the mathematical equations, linearization methods often result in underestimation of macroparameters [7].  Another aspect related to the linearization methods is that the later time points have a high influence on the slope of the linearization and therefore govern the eventual parameter estimation. When blood samples of early time points are used to calibrate and metabolite correct the non-invasive input curve instead of late time points, this might affect the absolute quantification and thus result in erroneous quantification. Nonetheless, the linearization methods use the AUC of the input function, as such small errors in estimating the input function, like estimating the peak activity levels, have less impact on the quantification.

For tracers that are not metabolised, in particular [^18^F]FDG, the bias was generally limited. For tracers that generate plasma metabolites, the non-invasive methods either overestimate (slope > 1.05) or underestimate (slope < 0.95) outcome parameters (< 5% deviation of the slope is considered to be due to noise), which indicates increased bias of the methods as compared to AIF derived quantification. Remarkably, the PBIF studies reported slopes of ~ 1.00, which is in contrast to IDIF and SIME. This suggests that the variation in bias might arise from difficulties measuring the true AIF, as the measured AIF suffers from delay and dispersion effects. As the PBIF is an average of measured AIF’s, the dispersion effects are incorporated in the curves, while for IDIF and SIME the true AIF is estimated without interference of dispersion effects and might thus represent values closer to the ground truth. Yet, in case of high repeatability and correlation, the non-invasive methods could still be employed to substitute the AIF if the bias remains constant.

Studies were included in which correlations were reported between results of non-invasive quantification methods and pharmacokinetic modelling with an AIF, but many studies did not report the slope and intercept of this correlation, and therefore it was not clear how well the values of the estimated parameters corresponded. Depending on the purpose of PET quantification, knowledge regarding inter-subject variability of the bias (e.g. slope, intercept) could be important for example when absolute quantification is necessary for treatment decision making. Therefore, it is highly recommended that future studies do not only report the correlation, but also the slope and intercept of the correlation.

Non-invasive kinetic modelling approaches need to be validated per tracer, per pathology and for each intervention. Any disease or pharmacological substrate could theoretically affect tracer kinetics (e.g. alterations in liver function affecting tracer metabolism, or alterations in kidney function affecting tracer clearance) and, therefore, performance of the non-invasive kinetic approaches. From a theoretical perspective, PBIF is most vulnerable by disease factors and interventions, especially in cases where the disease or intervention strongly affects tracer metabolism and plasma clearance. IDIF and SIME should be less affected by pathology. The IDIF is directly extracted from a blood pool in the scan and should therefore be affected in the same manner by pathology or intervention as the AIF.

So far, the PBIF has mainly been individualized using variables related to body composure (e.g. body weight, body height, body surface area, body mass index, lean body mass) and injected dose. Some studies have shown that measurements related to heart rate and blood pressure correlate better with measurements of tracer concentrations in blood [11, 37], and therefore correction using these variables might enhance the correspondence of the PBIF.

In this study, we considered the AIF as gold standard. However, many experimental factors influence the accuracy of the AIF, like the use of manual or automatic sampling, sensitivity of the blood monitor, the number of blood samples, the time points of the blood samples or the accuracy of the measurement of the manual samples. While manual sampling of the AIF is not ideal as it has a lower temporal resolution as compared to the automatic sampling, the automatic sampling suffers from dispersion effects. However, dispersion effects influence mostly the estimation of the microparameters, but not the macroparameters, and therefore the effects of dispersion are often negligible for PET quantification. Furthermore, the AIF is also depended on the tracer injection procedure. Manual tracer injection can introduce confounders (like variations in injection speed, injection pressure, injection volume, volume of flushing) which influence the shape of the measured AIF’s. This issue could be easily solved with an automated injection system for tracer administration. In addition, there is variation in automated injection regarding bolus injection and dynamic injection. A bolus injection is considered to lead to better estimations of the microparameters, whereas no differences between these 2 injection schemes should arise for macroparameter estimation. The major source of errors in the AIF, however, is usually the assessment of the tracer parent fraction. Not only the assessment of the parent fraction itself is prone to errors due to low count rates, but also the time resolution of the plasma parent curve is usually poor. Due to the time-consuming procedure of the metabolite analysis, the number of plasma samples that can be measured is usually limited, especially for tracers labelled short-lived isotopes like ^11^C. Therefore, some concerns regarding the AIF as a gold standard can be raised as well.

## Conclusion

Several studies have shown good correspondence between quantitative outcome measures derived using PBIF, IDIF or SIME and corresponding outcome measures obtained using arterial sampling. However, the need for arterial blood samples eliminates the potential benefits of these methods and hampers clinical utilization. A limited number of studies (with small sample sizes) suggest that IDIF and SIME-based methods may have the potential to omit any arterial sampling. In particular for PET tracers that are not metabolised, the use of IDIF and SIME without any blood data was promising, and especially the combination of IDIF and SIME. However, given the limited number of studies with small sample sizes, these methods require further validation in various patient groups before they routinely can be implemented. Future studies that investigate the implementation of non-invasive PET quantification should not only report correlation coefficients, but also parameters that reflect the bias of the parameter estimates, such as slope, intercept and ICC, as this is crucial for the assessment whether the technique could be employed to substitute the AIF. We still may not be there in the clinic, but path forward becomes more clear.

## Supplementary Information

Below is the link to the electronic supplementary material.

Supplementary file1 (DOCX 982 KB)

## Abbreviations

1T2k: Reversible one tissue compartment model
2T3k: Irreversible two tissue compartment model
2T4k: Reversible two tissue compartment model
AIF: Arterial input function
AUC: Area under the curve
BP_ND_: Binding potential
CMR_glc_: Cerebral glucose metabolic rate
DVR: Distribution volume ratio
EHR: Electronic health record
GM: Grey matter
ICA: Independent component analysis
ICC: Intraclass correlation coefficient
IDIF: Image derived input function
K_i_: Net influx rate
LEGA: Likelihood estimation in graphical analysis
MRI: Magnetic resonance imaging
pAIF: Predicted arterial input function
pAUC: Predicted area under the curve
PBIF: Population-based input function
PET: Positron emission tomography
PRISMA-DTA: Preferred reporting items for a systematic review and meta-analysis for diagnostic test accuracy
PVC: Partial volume correction
PWC: Pairwise correlation
QUADAS-2 tool: Quality Assessment of Diagnostic Accuracy Studies-2 tool
ROI: Region of interest
SIME: Simultaneous estimation of the input function
SUV: Standardized uptake value
TAC: Time activity curve
TRV: Test-rest variability
V_T_: Volume of distribution
WM: White matter
