# Supplementary material for: Non-invasive kinetic modelling approaches for quantitative analysis of brain PET studies
Source: Eur J Nucl Med Mol Imaging. 2023 Jan 18;50(6):1636–50. doi: 10.1007/s00259-022-06057-4 (PMC10119247; doi:10.1007/s00259-022-06057-4)
Supplement: Supplementary file 1 — Supplementary file1 (DOCX 982 KB) [file 259_2022_6057_MOESM1_ESM.docx]

Supplementary information Table 1: Methodological aspects for generation of PBIF

| Study | Tracer | Blood calibration | Sample | Test set | PBIF standardization | PBIF individualisation |
| --- | --- | --- | --- | --- | --- | --- |
| Roccia, 2019[11] | [^18^F]FDG | 1 arterial sample, 40 min | 49 (HC, MCI, AD, but not specified how many per group) | Not specified | 3-exponential model, height, weight | Scaled with blood sample |
| Takikawa, 1993[7] | [^18^F]FDG | 2 arterial samples, 10 + 45 min | 3 (of 13) HC  7 (of 21) movement disorder | 10 (of 13) HC  14 (of 21) movement disorder | AIFs were delay corrected, divided by subject body weight, and then averaged | Scaled with blood samples |
| McGinnity, 2018[13] | [^18^F]GE179 | 1 arterial sample, 90 min | 9 HC  11 epilepsy | LVOO | Injected dose, weight, and age were used to normalise AIF. Time shift was performed and the median was calculated to generate PBIF. | Scaled with blood samples |
| Lavisse, 2015[14] | [^18^F]DPA714 | 1 arterial sample, 40 min | 10 HC | LVOO | AIFs were time shifted and averaged | Scaled with blood samples |
| Lavisse, 2015[14] | [^18^F]DPA714 | 7 venous samples, 5, 10, 20, 40, 60, 70 and 90 min | 10 HC | LVOO | AIFs were time shifted and averaged | Scaled with blood samples |
| Mabrouk, 2017[15] | [^18^F]FEPPA | 5 arterial samples, 12, 30, 45, 60 and 90 min  2 arterial samples, 52 + 75 min  1 arterial sample, 75 min | 8 HC  8 PD  8 AD | 39 HC  16 PD  18 AD | AIFs corrected for body weight and injected dose, time shifted, and averaged. | Scaled with blood samples |
| Rissanen, 2015[16] | [^11^C]TMSX | 2 arterial samples, 9+23 min | 7 HC  12 MS  9 PD | LVOO | AIFs corrected for body weight and injected dose, time shifted, and averaged. | 1) Body surface area  2) Using 2 arterial samples |
| Rissanen, 2015[16] | [^11^C]TMSX | - | 7 HC  12 MS  9 PD | LVOO | AIFs corrected for body weight and injected dose, time shifted, and averaged. | Body surface area |
| Zanotti-Fregonara, 2013[17] | [^18^F]FMPEP-d2 | 2 arterial samples, 15 + 60 min | 42 HC | LVOO | AIFs corrected for body weight and injected dose, time shifted, and averaged. | Scaled with blood samples |
| Takikawa, 1994[12] | [^18^F]FDOPA | 2 arterial samples, 10 + 40 min | 10 subjects from different study | 12 HC  12 PD | Standardized metabolite corrected TAC | Scaled with blood samples |

*HC = healthy control, MCI = mild cognitive impairment, AD = Alzheimer’s disease, AIF = arterial input function, PD = Parkinson’s disease, MS = multiple sclerosis, TAC = time activity curve, MDD = major depressive disorder, LVOO = leave-one-out

Supplementary information Table 2: Methodological aspects for generation of IDIF

| Study | Tracer | Study population | ROI used | ROI defined | Blood calibration | Scanner | Spatial resolution | Time scan | Corrections |
| --- | --- | --- | --- | --- | --- | --- | --- | --- | --- |
| Zhou 2012[18] | [^18^F]FDG | 16 HC (out of 29 that were used in Chen 1998) | Whole brain vasculature | extracted based on the different monotonicity between the input and output function curves. | 1 arterial sample 30 min | 951/31 ECAT Siemens | 9.5 mm FWHM | 60 m | Spillover, PV |
| Huisman 2012[23] | [^18^F]FDG | FDG 9 HC | CA | Four hottest pixels per plane, ten planes | 3 arterial samples  5, 7.5, and 9 min | HRRT Siemens/CTI | 3 mm | 60 min FDG | - |
| Zhou 2011[24] | [^18^F]FDG | 16 HC | CA | Carotid artery voxels based on the shape of their TACs | 2 venous samples 9 + 25 min | 951/31 ECAT Siemens | 9.5 mm FWHM | 60 min | Method of Chen with 2 venous samples |
| Chen 1998[8] | [^18^F]FDG | 26 HC | CA | > PM p = threshold (0-1) m = highest pixel value | 3 venous samples  mid timepoint last frames | 951/31 ECAT Siemens | 9.5 mm FWHM | 60 min | Linear least square method based on three TACs (𝑐𝑚𝑒𝑎𝑝, cp and ct) |
| Chen 2007[25] | [^18^F]FDG | 22 HC (same subjects in Chen 1998) | CA | ICA | 3 venous samples mid timepoint last frames | 951/31 ECAT Siemens | 9.5 mm FWHM | 60 min | VEN-ICA = Linear least square method based on three TACs (𝑐𝑚𝑒𝑎𝑝, cp and ct)  local frame-wise maximal activity from the ICA identified blood vessel region over the first 30 min interval |
| Croteau 2010[26] | [^18^F]-FDG | 6 HC | CA | value of the four highest adjacent pixels for each plane | - | Gemini TF Philips | Size of isotropic voxel 2 mm | 80m | PV  spill-in effects |
| Galovic, 2021[20] | [^18^F]GE179 | 10 HC | CA | TOF-MRA scans | 1 venous sample: 62 min | GE Discovery 690 PET/CT | n.r. | 90 min | PVC |
| Mabrouk 2014[27] | [^18^F]FEPPA | 18 HC | CA | - Mean of the ten highest pixel activities per plane in the lowest 36 planes.  -Independent component analysis | 2 arterial samples: 1.5 min 15 min | 3D HRRT Siemens PET | 2.5 mm | 120 m | **-** |
| Zanderigo 2018[28] | [^11^C]Arachidonic acid | 11 HC | CA | Chen 1998 | - | Siemens Biograph mCT |  | 60 m | Correction SO/SI effects, Chen 1998, based on phantom data |
| Mertens, 2021[22] | [^18^F]JNJ-64413739 | 11 HC | CA | TOF-MRA | 22 arterial samples: 10, 20, 30, 40, 50, 60, 70, 80, 90, 100, 110, 120, 150, 180, 240, 300, 600, 1200, 1800, 2400, 3600, and 4800 sec | GE PET-MR | 7 mm FWHM | 90 min | PVC |
| Schain 2013[29] | [^11^C]Flumazenil | 6 HC | CA | - PWC - Mourik - Chen | 5 arterial samples, 36, 42, 48, 54, 60 min | HRRT Siemens | 1.5 mm FWHM | 63 min | Spill-out early frames (<3 min), scale tail (>3 min) using manual samples |
| Schain 2013[29] | [^11^C]AZ10419369 | 6 HC | CA | - PWC - Mourik - Chen - Naganawa | 1 Venous sample, 90 min | HRRT Siemens | 1.5 FWHM | 93 min | Spill-out early frames (<3min), scale tail (>3 min) using manual samples |
| Bahri 2017[30] | [^18^F]UCB-H | 4 HC | CA | Seeding region based on the highest local maximum to create a ‘subset’ mask | 3 arterial samples:  35, 60, 90 min | ECAT HR+ Siemens/CTI |  | 100 m | - |
| Islam 2017[19] | [^15^O]H_2_O | 33 subjects with CVD (15 unilateral stenosis 2 bilateral stenosis 16 moyamoya) | CA | Most intense 30 voxels of the 12 slices at skull base. 3D TOF MRI images as co-registration | - | ADVANCE Whole Body PET GE Medical Systems | 4.6-5.7 mm transaxial x 4.0-5.3 mm axial | 3 min | - |
| Vestergaard, 2021[21] | [^15^O]H_2_O | 12 HC  7 SCD | CA | TOF-MRA  >50% of max activity | - | 3 T Siemens Biograph mMR hybrid PET/MR |  | 4 min | - |

*HC = healthy control, CA = carotid arteries, FWHM = full width half max, PVC = partial volume correction, TAC = time activity curve, ICA = independent component analysis, CVD = cerebrovascular disease, SCD = steno-occlusive cerebrovascular disease, TOF-MRA = time of flight magnetic resonance arteries, PWC = pairwise correlation

Supplementary information Table 3: Methodological aspects for generation of SIME

| Study | Tracer | Blood calibration | Study Population | ROIs used | EHR parameters |
| --- | --- | --- | --- | --- | --- |
| Roccia, 2019[11] | [^18^F]FDG | 1 arterial sample, 40 min | 49 (HC, MCI, AD, not specified how many per group) | Prefrontal cortex, cingulate, hippocampus, parahippocampus, cerebellum GM  3E-AIF  PK-AIF | - |
| Ogden, 2010[31] | [^18^F]FDG | 1 arterial sample, 40 min | 9 not specified | Cerebellum, cingulate, hippocampus, prefrontal cortex, parahippocampus | - |
| Wong, 2001[33] | [^18^F]FDG | 2 venous samples, 30 and 60 min | 2 HC  1 epilepsy | GM, WM, whole brain | - |
| Roccia, 2019[11] | [^18^F]FDG | - | 49 (HC, MCI, AD, not specified how many per group) | Prefrontal cortex, cingulate, hippocampus, parahippocampus, cerebellum GM  3E-AIF  PK-AIF | pAIF40: TACsum, red blood cell distribution width, injected dose, triglycerides  pAUC: TACsum, red blood cell distribution width, injected dose, body surface area, ColdGlu2, estimated total plasma volume, estimated resting metabolic rate |
| Schain, 2018[34] | [^11^C]PBR28 | Original AIF | 15 HC  21 AD | Thalamus, caudate, putamen, ventral diencephalon, brainstem, cerebellar cortex, superior frontal cortex, middle frontal cortex, inferior frontal cortex, orbitofrontal cortex, precentral cortex, paracentral cortex, superior parietal cortex, inferior parietal cortex, supramarginalgyrus, precuneus, postcentral gyrus, medial occipital cortex, lateral occipital cortex, lingual cortex, superior temporal cortex, middle temporal cortex, inferior temporal cortex, fusiform,hippocampus, entorhinal cortex, parahippocampal gyrus, anterior cingulate, posterior cingulate, insula | - |
| Schain, 2018[34] | [^11^C]PBR28 | - | 15 HC  21 AD | Thalamus, caudate, putamen, ventral diencephalon, brainstem, cerebellar cortex, superior frontal cortex, middle frontal cortex, inferior frontal cortex, orbitofrontal cortex, precentral cortex, paracentral cortex, superior parietal cortex, inferior parietal cortex, supramarginalgyrus, precuneus, postcentral gyrus, medial occipital cortex, lateral occipital cortex, lingual cortex, superior temporal cortex, middle temporal cortex, inferior temporal cortex, fusiform,hippocampus, entorhinal cortex, parahippocampal gyrus, anterior cingulate, posterior cingulate, insula | - |
| Bartlett, 2019[35] | [^11^C]CUMI | 1 arterial sample, 30 min | 19 not specified | cerebellar GM, midbrain, amygdala, dorsal caudate, hippocampus, temporal  cortex, ventral striatum | - |
| Zanderigo, 2015[36] | [^11^C]CUMI | 1 arterial sample, 60 min | 9 HC  5 MDD | 3 ROIs out of 5 clusters extracted. Here, we automatically select the regions for SIME using a data-driven voxelbased algorithm that determines five subject-specific regions via kmeans clustering of gray and white matter voxels |  |
| Bartlett, 2019[35] | [^11^C]CUMI | 1 venous sample, 90 min | 19 not specified | cerebellar GM, midbrain, amygdala, dorsal caudate, hippocampus, temporal  cortex, ventral striatum | - |
| Zanderigo, 2015[36] | [^11^C]CUMI | - | 9 HC  5 MDD | 3 ROIs out of 5 clusters extracted. Here, we automatically select the regions for SIME using a data-driven voxelbased algorithm that determines five subject-specific regions via kmeans clustering of gray and white matter voxels | Computed blood anchor |
| Ogden, 2010[31] | [^11^C]DASB | 1 arterial sample, 50 min | 25 not specified | Cerebellum GM, midbrain, amygdala, dorsal caudate, hippocampus, temporal lobe, ventral striatum | - |
| Bartlett, 2019[35] | [^11^C]DASB | 1 arterial sample, 80 min | 18 not specified | cerebellar GM, midbrain, amygdala, dorsal caudate, hippocampus, temporal  cortex, ventral striatum | - |
| Bartlett, 2019[35] | [^11^C]DASB | 1 venous sample, 100 min | 18 not specified | cerebellar GM, midbrain, amygdala, dorsal caudate, hippocampus, temporal  cortex, ventral striatum | - |
| Mikhno, 2015[37] | [^11^C]DASB | - | 95 not specified | Cerebellum GM, midbrain, amygdala, dorsal caudate, hippocampus, temporal lobe, ventral striatum  PK-AIF | pAIF50: cerebellum TAC sum, sum of the curves of total radioactivity in plasma, hear rate after PET scan * sum of the curves of total radioactivity in plasma  pAUC: sum of the curves of total radioactivity in plasma, cerebellum TAC sum, body surface area * pulse pressure post PET scan |
| Ogden, 2010[31] | [^11^C]WAY-100635 | 1 arterial sample, 20 min | 7 not specified | Cerebellum WM, hippocampus, temporal lobe, occipital lobe, anterior cingulate | - |
| Sari, 2018[38] | [^11^C]SB201745 | - | 6 HC | Cerebellum, parietal cortex, hippocampus, striatum  SIME contrained has fixed k1/k2  SIME original not fixed k1/k2 | IDIF |
| Bartlett, 2019[35] | [^11^C]ABP688 | 1 arterial sample, 12 min | 10 not specified | Zanderigo 2010 & 2015 | - |
| Bartlett, 2019[35] | [^11^C]ABP688 | 1 venous sample, 12 min | 10 not specified | Zanderigo 2010 & 2015 | - |
| Zanderigo, 2018[32] | [^11^C]Harmine | 1 arterial sample, 20 min | 5 HC | Cerebellar GM, dorsal caudate, dorsal putamen, entorhinal cortex, hippocampus, temporal lobe, thalamus | - |
| Ogden, 2010[31] | [^11^C]BTA | 1 arterial sample, 40 min | 10 not specified | Cerebellum, cingulate, hippocampus, prefrontal cortex, parahippocampus | - |

*AD = Alzheimer’s disease, HC = healthy control, MCI = mild cognitive impairment, AIF = arterial input function, GM = grey matter, WM = white matter, AUC = area under the curve, pAUC = predicted AUC, pAIF = predicted AIF, TAC = time activity curve, MDD = major depressive disorder, ROI = region of interest, CVD = cerebrovascular disease

Supplementary information Table 4: Less-invasive kinetic modelling approaches separated per tracer.

| PET tracer | PBIF arterial calibration | PBIF arterio-venous calibration | PBIF venous calibration | PBIF without calibration |  | IDIF arterial calibration | IDIF venous calibration | IDIF without  calibration | SIME arterial calibration | SIME venous calibration | SIME without calibration |
| --- | --- | --- | --- | --- | --- | --- | --- | --- | --- | --- | --- |
| [^11^C]ABP688 |  |  |  |  |  |  |  |  | • | • |  |
| [^11^C]Arachidonic |  |  |  |  |  |  |  | • |  |  |  |
| [^11^C]AZ10419369 |  |  |  |  |  | • |  |  |  |  |  |
| [^11^C]BTA |  |  |  |  |  |  |  |  | • |  |  |
| [^11^C]CUMI |  |  |  |  |  |  |  |  | • | • |  |
| [^11^C]DASB |  |  |  |  |  |  |  |  | • | • | • |
| [^11^C]Flumazenil |  |  |  |  |  | • |  |  |  |  |  |
| [^11^C]Harmine |  |  |  |  |  |  |  |  | • |  |  |
| [^11^C]PBR28 |  |  |  |  |  |  |  |  | • |  | • |
| [^11^C]PiB |  |  |  |  |  |  |  |  |  |  |  |
| [^11^C]SB201745 |  |  |  |  |  |  |  |  |  |  | • |
| [^11^C]TMSX | • |  |  | • |  |  |  |  |  |  |  |
| [^11^C]WAY-100635 |  |  |  |  |  |  |  |  | • |  |  |
| [^15^O]H_2_O |  |  |  | • |  |  |  | • |  |  |  |
| [^18^F]DPA714 | • |  | • |  |  |  |  |  |  |  |  |
| [^18^F]DPA713 | • |  |  |  |  |  |  |  |  |  |  |
| [^18^F]FDG | • | • |  |  |  | • | • | • | • | • | • |
| [^18^F]FDOPA | • |  |  |  |  |  |  |  |  |  |  |
| [^18^F]FEPPA | • |  |  |  |  | • |  |  |  |  |  |
| [^18^F]FLT |  |  |  |  |  |  |  | • |  |  |  |
| [^18^F]FMPEP-d_2_ | • |  |  |  |  |  |  |  |  |  |  |
| [^18^F]GE179 | • |  |  |  |  |  | • |  |  |  |  |
| [^18^F]PK209 |  |  |  | • |  |  |  |  |  |  |  |
| [^18^F]UCB-H |  |  |  |  |  | • |  |  |  |  |  |
| [^18^F]JNJ-64413739 |  |  |  |  |  | • |  |  |  |  |  |

** IDIF = Image Derived Input Function, PBIF = Population Based Input Function, SIME = Simultaneous Estimation of the Input Function*
